# Association of Individual Non-Steroidal Anti-Inflammatory Drugs and Chronic Kidney Disease: A Population-Based Case Control Study

**DOI:** 10.1371/journal.pone.0122899

**Published:** 2015-04-16

**Authors:** Ylenia Ingrasciotta, Janet Sultana, Francesco Giorgianni, Andrea Fontana, Antonio Santangelo, Daniele Ugo Tari, Domenico Santoro, Vincenzo Arcoraci, Margherita Perrotta, Luisa Ibanez, Gianluca Trifirò

**Affiliations:** 1 Department of Clinical and Experimental Medicine, University of Messina, Messina, Italy; 2 Unit of Biostatistics, IRCCS "Casa Sollievo della Sofferenza", San Giovanni Rotondo (FG), Italy; 3 Caserta Local Health Service, Caserta, Italy; 4 Fundació Institut Català de Farmacologia, WHO Collaborating Centre for Research and Training in Pharmacoepidemiology, Department of Pharmacology, Therapeutics and Toxicology, Institut Català de la Salut, Universitat Autònoma de Barcelona, Barcelona, Spain; University of São Paulo School of Medicine, BRAZIL

## Abstract

**Background:**

Non-steroidal anti-inflammatory agents (NSAIDs) are known to be associated with renal damage. No clear evidence exists regarding differential risk of chronic kidney disease (CKD), specifically, across various NSAIDs.

**Aim:**

The aim of this population-based case-control study was to evaluate the association between use of individual NSAIDs and risk of CKD in a general population of Southern Italy.

**Methods:**

A nested case-control study was carried out using the general practice Arianna database, identifying incident CKD patients as cases and matched controls from 2006 to 2011. The date of first CKD diagnosis was defined as the index date (ID). Conditional logistic regressions were performed to estimate the risk of CKD associated with NSAIDs by class and individual drugs as compared to non-use during different time windows (within one year, six or three months prior to ID), with the latter being defined as current users. Among current users, the effect of cumulative exposure to these drugs was evaluated.

**Results:**

Overall, 1,989 CKD cases and 7,906 matched controls were identified. A statistically significant increase in the risk of CKD was found for current users of oxicams (adjusted OR: 1.68; 95% CI: 1.15-2.44) and concerning individual compounds, for ketorolac (adj. OR: 2.54; 95% CI: 1.45-4.44), meloxicam (adj. OR: 1.98; 95% CI: 1.01-3.87) and piroxicam (adj. OR: 1.95; 95% CI: 1.19-3.21).

**Conclusions:**

The risk of CKD varies across individual NSAIDs. Increased risk has been found for ketorolac, which may precipitate subclinical CKD through acute renal damage, and long-term exposure to oxicams, especially meloxicam and piroxicam.

## Introduction

Chronic kidney disease (CKD) represents an important cause of morbidity and mortality worldwide [1, 2]. The prevalence of CKD has been continuously increasing in recent decades. The prevalence of CKD seems to be comparable globally and tapers off at approximately 9–12% in the USA, Australia [3] and Europe, particularly in the elderly population [4]. Apart from ethnicity, slight differences in prevalence depend also upon the method of CKD identification, the formula for estimating the glomerular filtration rate (GFR), and the setting of the study.

The main underlying factors driving the progressively increasing prevalence of CKD are the ageing of global populations [5], the global epidemic of type 2 diabetes mellitus [6] and other co-morbidities such as hypertension [3, 7–9]. In addition to this, several drugs which are widely used in general population [10] such as non-steroidal anti-inflammatory drugs (NSAIDs) can affect renal function [11, 12]. NSAIDs are commonly known to cause acute kidney injury (AKI) through multiple mechanisms, accounting for 16% of all drug-related renal failure [13]. Besides producing a reversible renal failure, NSAIDs are known to cause acute interstitial nephritis (AIN) with hematuria, proteinuria and flank pain [14] as well as acute tubular necrosis (ATN). Rare mechanisms include renal vasculitis and acute papillary necrosis [15].

It is known that NSAID use, in general, can also increase the risk of accelerated CKD progression through both non-immunologic and immunologic mechanisms. Immunological reactions that develop during the acute phase may continue to take place after the initial kidney insult occurs [16]. In general, recurrent episodes of NSAID-related AKI may lead to CKD or chronic exposure to NSAIDs may worsen unrecognized AIN that can evolve into chronic interstitial nephritis (CIN) with associated interstitial fibrosis or chronic papillary necrosis.

NSAIDs are known to cause acute adverse effects on the kidney, but chronic renal effects associated with NSAIDs are less well-described. Phenacetin is the only NSAID known to be related to chronic renal effects because it was withdrawn from the market due to renal papillary necrosis leading to chronic kidney disease [17].

The role of half-lives as a basis for nephrotoxicity has been studied. Adams et al. proposed that NSAIDs with longer half-lives are more likely to cause nephrotoxicity because of sustained prostaglandin inhibition leading to a sustained reduction in renal blood flow, whereas with short-acting NSAIDs the kidney may be able to recover better between doses [18]. To explore the association between NSAID use and new onset of CKD is challenging as CKD may not be immediately apparent and can be preceded by subclinical renal damage [19]. Most of the previous studies investigated the risk of acute renal failure [20–22], rather than chronic kidney disease in association with NSAIDs exposure. Other studies focused on the progression of CKD rather than its occurrence [23–25].

To date, the differential association between individual NSAIDs and CKD risk has not been yet investigated in detail.

The aim of this population-based study was to evaluate and compare the association between the use of individual NSAIDs and risk of CKD in a general population of Southern Italy.

## Methods

### Data Source

Data were extracted from the Arianna database, a general practice database that was set up by Caserta Local Health Unit from Southern Italy in 2000. Arianna contains clinical and demographic data from almost 300,000 inhabitants living in the area of Caserta and who are registered with roughly 200 general practitioners (GPs).

The database also contains information on prescribed drugs (registered using the Anatomical and Therapeutic Chemical code) reimbursed by the National Health Service, along with their indication of use. Hospital admissions and medical procedures are registered using the ninth edition of International Classification of Diseases, Clinical Modification (ICD-9 CM). Previously published research shows the Arianna database to be a reliable data source for pharmacoepidemiological research [10, 26–32].

All participating GPs record data during their routine clinical practice using dedicated software. This data is anonymized and transferred monthly to the Arianna database. This database can be linked to a hospital discharge registry and other administrative registries within the same catchment area through a unique anonymous patient identifier. The quality of the collected data is checked regularly as described elsewhere and only GPs who submit data meeting pre-defined quality criteria are included [10]. A total of 123 GPs and 158,510 of their patients were included in this study.

### Ethics Statement

This is an observational, retrospective, non-interventional study. According to a by-law on the classification and implementation of observational drug-related research, as issued by the Italian National Drug Agency (an entity belonging to the Italian Ministry of Health), the present study does not require approval by an Ethics Committee in Italy (Italian Drug Agency note of 3^th^ August 2007) [33].

None of the co-authors are among the general practitioners involved in data collection within the Arianna database. The use of Arianna data was made possible through collaboration between the Caserta Local Health Service, represented by DUT and the University of Messina, represented by GT. All data extracted from the database are fully anonymized before the authors received the data set and carried out the data analysis.

### Study population

All the CKD-free patients who were registered in the Arianna database and with at least one year database history were eligible to be included in the study. The study period ran from 2006 to 2011.

### Case ascertainment

We identified as cases all patients with an incident diagnosis of CKD occurring during the study period. CKD diagnoses were identified by searching for specific ICD9-CM codes among either primary/secondary causes of hospital admissions, hospital procedures or indication of use associated to the CKD-related drug prescriptions, as described previously [10]. The index date (ID) was defined as the date of first CKD diagnosis.

### Control selection

**Fig 1:**
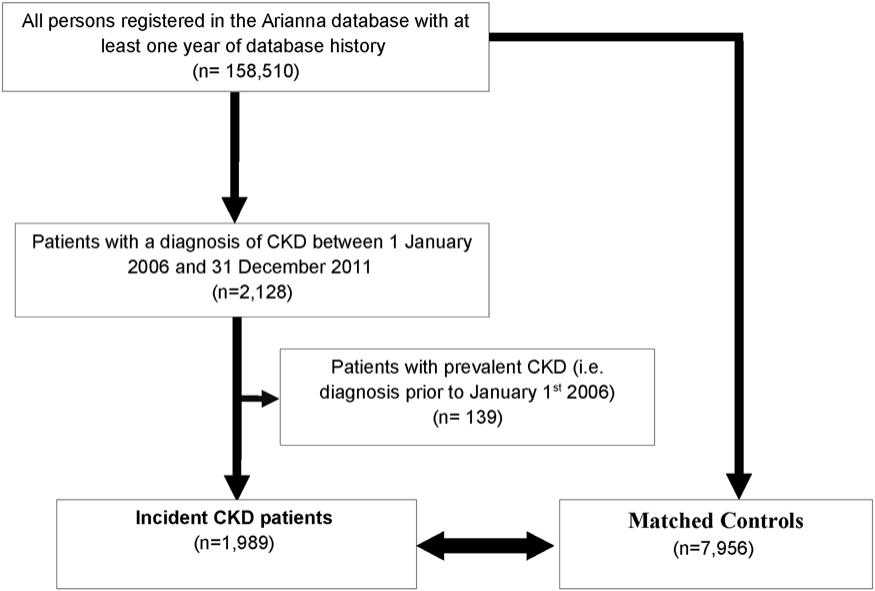
Selection of cases and controls.

From the same source population, for each case, up to 4 CKD-free controls (which were in follow-up at the index date of the case) were randomly selected and matched by age (± 3 years) and sex. The same index date of the case was assigned to the matched controls (Fig 1).

### Drug exposure

Non-steroidal anti-inflammatory drugs (NSAIDs) were the exposure of interest, which was evaluated using prescription data. For each prescription, duration of use was calculated on the basis of the prescribed amount of active principle and the defined daily dose (DDD).

NSAID exposure was evaluated overall and by individual compounds and classes, as categorized below: acetic acid derivatives and related substances (ATC: M01AB*), oxicams (M01AC*), propionic acid derivatives (M01AE*), other anti-inflammatory and antirheumatic agents (non-steroids) (M01AX*), coxibs (M01AH*) and salicylic acid and derivatives (N02BA*). The exposure to NSAIDs was always assessed prior to the index date. Use of more than one NSAID was evaluated separately and mutually exclusive categories of individual NSAID exposure compared to non-exposure were considered. The exposure to NSAIDs was evaluated within three different observation periods (*time windows*): 90, 180, and 365 days prior to the Index Date. The number of cases and controls using one or more than one NSAID over the different time windows was reported in S1 Table. Patients receiving at least one NSAID prescription in the first time window (within 90 days prior to the index date) were defined as “current users” and were investigated in more detail as more strictly related in time to the onset of CKD.

To explore the effect of NSAID treatment duration of use on CKD risk, *“cumulative exposure to NSAIDs”* was categorized in three classes among current users: less than or equal to 90 days, between 91 and 180 days, more than 180 days. For each prescription, the duration of exposure was estimated by dividing the total amount of active principle in each prescription with NSAID-specific defined daily dose (DDD). The estimated number of days of NSAID exposure for each prescription issued during the observation period was summed up.

### Covariates

As covariates we identified all the potential risk factors for CKD, including co-morbidities (i.e. malignant neoplasm, ischemic heart disease, diabetes mellitus, liver disease, gout, dyslipidemia, hypertension, cerebrovascular disease, lupus erythematosus systemic (LES), amyloidosis, vasculitis, myeloma, polycystic kidney disease), and prior use of known nephrotoxic drugs other than NSAIDs (e.g. aminoglycosides, gold preparations, and lithium) [34]. All the covariates were assessed any time prior to the index date.

### Statistical analysis

Demographic and clinical characteristics of cases and matched controls were compared using the chi-square test and two sample t-test for categorical and continuous variables respectively. The assumption of normal distribution was tested using Shapiro-Wilks and Kolmogorov-Smirnov tests.

To estimate the risk of CKD associated with individual NSAIDs as compared to non-use of NSAIDs, univariate and multivariate conditional logistic regression models were performed. Multivariate models included all those covariates which were significantly associated with CKD risk at the univariate models. The risk of CKD was reported as adjusted odds ratio (adj. OR) along with their 95% confidence intervals (95% CI).

Moreover, the risk of CKD for each additional cumulative month of NSAIDs exposure (based on DDD) was evaluated any time and within one year prior to ID, including the number of months of therapy as a continuous variable in the conditional logistic regression model. Such analysis was performed for NSAIDs, as a whole class, and for oxicams.

To statistically evaluate whether hypertension and diabetes mellitus act as effect modifiers of the association between exposure to drugs and risk of CKD, their interaction terms were included into the models and p-values were derived from the test of fixed effects.

A two-sided p-value of <0.05 was considered as the threshold for statistical significance. All the analyses were performed using SAS Release 9.3 (SAS Institute, Cary, NC).

## Results

Overall, 2,128 patients with a diagnosis of CKD were identified during the years 2006–2011 in a general population of Southern Italy. Of these, 1,989 (1.3%) who were newly diagnosed during the follow-up were included in the study. Cases were matched by age, sex and index date to 7,906 controls (Fig 1). In Table 1 the characteristics of cases and controls are reported. Cases and controls were equally distributed between males and females and over 75% of them were older than 65 years (Table 1). As compared to controls, CKD patients were significantly more affected by malignant neoplasm, ischemic heart disease, diabetes mellitus, liver disease, gout, dyslipidemia, hypertension, cerebrovascular disease and other CKD-related diseases as well as received more frequently nephrotoxic drugs other than NSAIDs (p = 0.013).

**Table 1:** Characteristics of incident CKD cases and matched controls.

	Cases^a^	Controls^a^	P-value
	N = 1,989 (%)	N = 7,906 (%)	
**Sex**			Matching factor
Men	980 (49.3)	3,900 (49.3)	
Women	1,009 (50.7)	4,006 (50.7)	
**Mean age (SD), y**	72.4 (13.4)	72.0 (13.4)	
**Age categories (y)**			Matching factor
<45	82 (4.1)	334 (4.2)	
45–64	385 (19.4)	1,563 (19.8)	
65–80	933 (46.9)	3,751 (47.4)	
>80	589 (29.6)	2,258 (28.6)	
**Mean duration of follow-up (SD), y**	5.5(1.1)	5.9(0.6)	
**Co-morbidities** ^b^			
Malignant neoplasm	212 (10.7)	479 (6.1)	<0.001
Ischemic heart disease	734 (36.9)	1,579 (20.0)	<0.001
Diabetes mellitus	725 (36.5)	1,257 (15.9)	<0.001
Liver disease	129 (6.5)	233 (2.9)	<0.001
Gout	541 (27.2)	581 (7.3)	<0.001
Dyslipidemia	624 (31.4)	1,470 (18.6)	<0.001
Hypertension	1,714 (86.2)	5,077 (64.2)	<0.001
Cerebrovascular disease	312 (15.7)	738 (9.3)	<0.001
Other CKD-related diseases^c^	55 (2.8)	49 (0.6)	<0.001
**Prior use of nephrotoxic drugs other than NSAIDs** ^b^ ^,^ ^d^	211 (10.6)	698 (8.8)	0.013

SD = Standard deviation; NSAID = non-steroidal anti-inflammatory drugs.

^a^ Values are reported as numbers (percentages), unless otherwise noted;

^b^ All the co-morbidities and the prior use of nephrotoxic drugs have been assessed any time prior to index date;

^c^ Other diseases which may increase the risk of CKD: Lupus Erythematosus Systemic (LES), amyloidosis, vasculitis, myeloma and polycystic kidney;

^d^ Sulfonamides, aminoglycosides, gold preparations, zoledronate, lithium and colistin.

**Fig 2:**
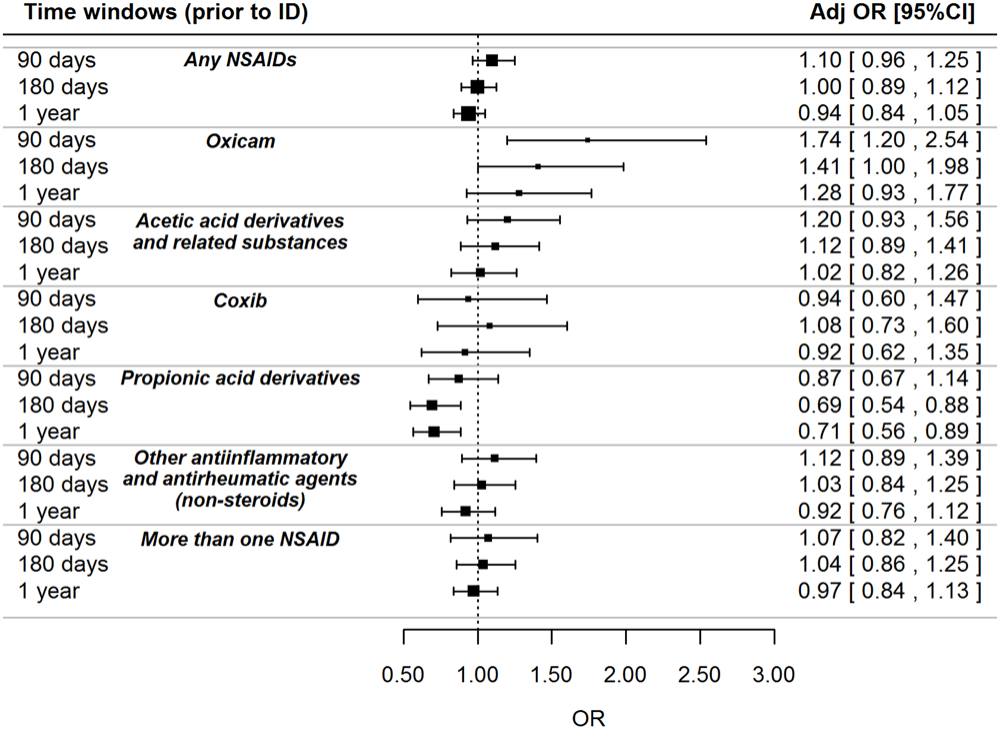
Association between NSAIDs and risk of CKD, stratified by classes and time windows. NSAID = non-steroidal anti-inflammatory drug; ID = index date; OR = odds ratio. OR was adjusted for malignant neoplasm, ischemic heart disease, diabetes mellitus, liver disease, gout, dyslipidemia, hypertension, cerebrovascular disease, lupus erythematosus systemic (LES), amyloidosis, vasculitis, myeloma, polycystic kidney, prior use of nephrotoxic drugs (other than NSAIDs). Oxicam: piroxicam, meloxicam, lornoxicam, tenoxicam; Acetic acid derivatives and related substances: aceclofenac, ketorolac, acemetacin, etodolac, diclofenac, diclofenac and combinations; Coxib: celecoxib and etoricoxib; Propionic and derivatives: ibuprofen, naproxen, ketoprofen, dexibuprofen, tiaprofenic acid, dexketoprofen, naproxen and esomeprazole; Other anti-inflammatory and anti-rheumatic agents, non-steroids: nimesulide, niflumic acid, acetylsalicylic acid, acetylsalicylic acid combinations excl. psycholeptics; More than one NSAID drug: at least two different drugs belonging to different classes prescribed during the observed time window.

**Fig 3:**
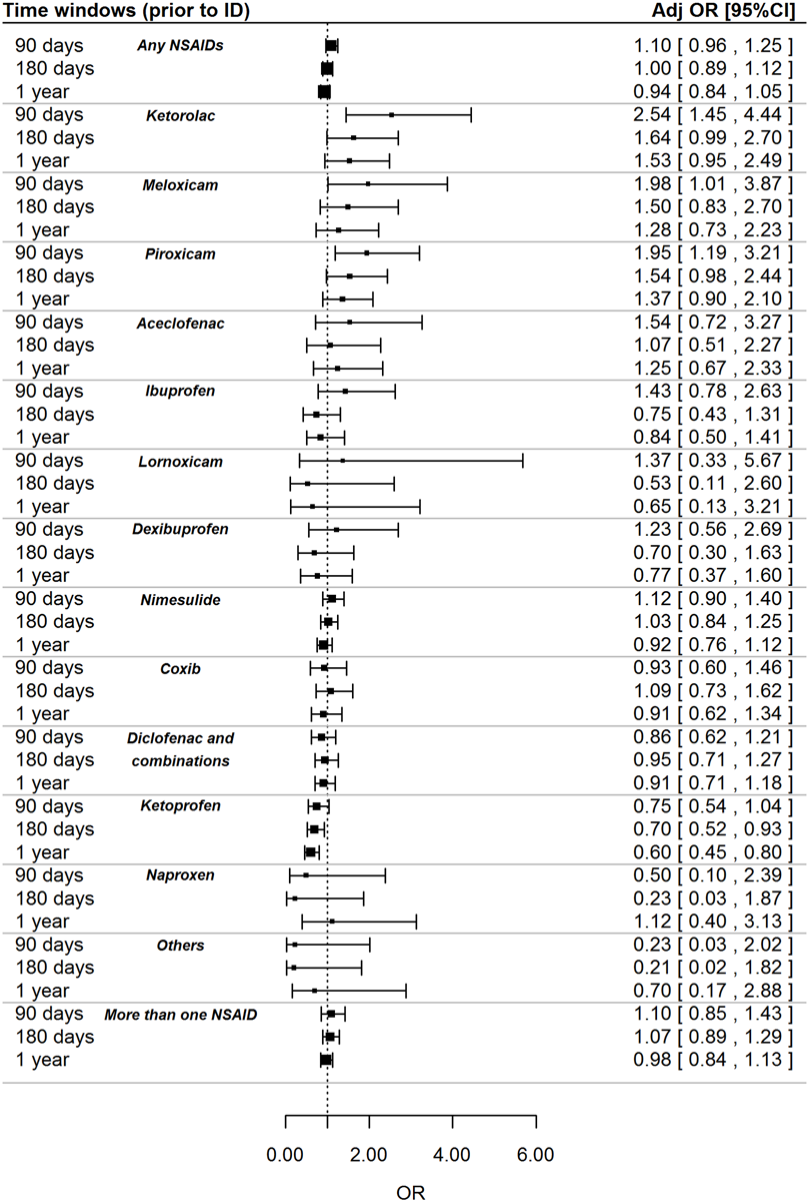
Association between individual NSAIDs and risk of CKD, stratified by time windows. NSAID = non-steroidal anti-inflammatory drug; ID = index date; OR = odds ratio. OR was adjusted for malignant neoplasm, ischemic heart disease, diabetes mellitus, liver disease, gout, dyslipidemia, hypertension, cerebrovascular disease, lupus erythematosus systemic (LES), amyloidosis, vasculitis, myeloma, polycystic kidney, prior use of nephrotoxic drugs (other than NSAIDs); Coxib: celecoxib and etoricoxib; Others: tenoxicam, aceclofenac, tiaprofenic acid, acetylsalicylic acid, acetylsalicylic acid combinations excl. psycholeptics, niflumic acid; More than one NSAID: at least two different drugs prescribed during the observed time window.

Results from multivariate models for NSAIDs as a whole and by class and individual compounds are reported in Figs 2 and 3 respectively. A statistically significant increase in the CKD risk was found for current users of oxicams (adj. OR: 1.74; 95% CI: 1.20–2.54; p = 0.004) and, concerning single ingredients, for current users of meloxicam (adj. OR: 1.98; 95% CI: 1.01–3.87; p = 0.046), piroxicam (adj. OR: 1.95; 95% CI: 1.19–3.21; p = 0.008) and ketorolac (adj. OR: 2.54; 95% CI: 1.45–4.44; p = 0.001). No statistically significant associations were found for larger time windows.

**Fig 4:**
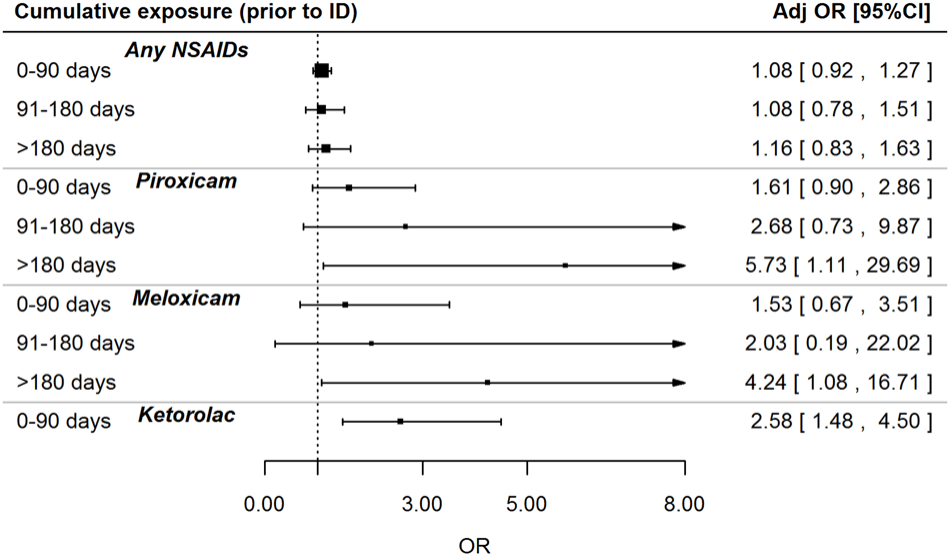
Effect of cumulative exposure to piroxicam, meloxicam, ketorolac and NSAIDs as a whole among current users on the CKD risk. NSAID = non-steroidal anti-inflammatory drug; ID = index date; OR = odds ratio. OR was adjusted for malignant neoplasm, ischemic heart disease, diabetes mellitus, liver disease, gout, dyslipidemia, hypertension, cerebrovascular disease, lupus erythematosus systemic (LES), amyloidosis, vasculitis, myeloma, polycystic kidney, prior use of nephrotoxic drugs (other than NSAIDs).

The association between exposure to piroxicam, meloxicam or ketorolac and adjusted risk of CKD was not significantly modified by the presence of hypertension or diabetes mellitus. The risk of CKD increased by 28% (adj. OR: 1.28; 95% CI: 1.08–1.52; p = 0.004) and by 11% (adj. OR: 1.11; 95% CI: 1.00–1.23; p = 0.041) for each additional cumulative month of therapy with meloxicam considering the time windows within one year prior and any time prior to ID, respectively (Table 2). Furthermore, the risk of CKD increased by 19% (adj. OR: 1.19; 95%CI: 0.98–1.46; p = 0.085) and by 13% (adj. OR: 1.13; 95% CI: 1.03–1.24; p = 0.007) for each additional cumulative month of therapy with piroxicam within one year prior and any time prior to ID, respectively (Table 2). Accordingly, as shown in Fig 4, among current users of piroxicam, the highest statistically significant increase of CKD risk was observed for cumulative exposure higher than 180 days (adj. OR: 5.73; 95% CI: 1.10–29.69; p = 0.038), as well as for meloxicam (adj. OR: 4.24; 95% CI: 1.08–16.71; p = 0.039). Ketorolac was always used for less than 3 months.

**Table 2:** CKD risk for each additional cumulative month of NSAIDs exposure, based on DDD, (as a class or individual compound), anytime and within one year prior to the index date.

	Adj. OR^a^ (95% CI)	P-value
**Any time prior to index date**		
Any NSAIDs	1.03 (1.01–1.05)	0.003
Piroxicam	1.13 (1.03–1.24)	0.007
Meloxicam	1.11(1.00–1.23)	0.041
**Within one year prior to index date**		
Any NSAIDs	1.07 (1.02–1.12)	0.004
Piroxicam	1.19 (0.98–1.46)	0.085
Meloxicam	1.28 (1.08–1.52)	0.004

NSAID = non-steroidal anti-inflammatory drug; ID = index date; OR = odds ratio.

^a^ Adjusted for malignant neoplasm, ischemic heart disease, diabetes mellitus, liver disease, gout, dyslipidemia, hypertension, cerebrovascular disease, lupus erythematosus systemic (LES), amyloidosis, vasculitis, myeloma, polycystic kidney, and nephrotoxic drugs (other than NSAIDs).

## Discussion

Our results suggest no statistically significant increase in the risk of CKD in association with NSAIDs as a whole class. A Spanish case-control study found that long-term use (6 months) of NSAIDs was not associated with an increased risk of end stage renal disease (ESRD) [35], in agreement with our study.

Previous studies investigating the relationship between exposure to individual NSAIDs and CKD risk reported conflicting results [36, 37]. Two previous reviews by McLaughlin et al. (1998) and Delzell et al. (1998) were unable to outline clear conclusions from this evidence [36, 37]. NSAIDs were found to be associated with an increase in the risk of CKD in two case-control studies [38, 39], but not in three cohort studies in healthy subjects [23–25, 40]. A case-control study found an association between the chronic use of acetylsalicylic acid in analgesic doses and the risk of CKD [41], although this association was not found in other two case-control studies (whose data on NSAID prescriptions were obtained by telephone interview) [38, 39], and three cohort studies [23–25], including only males [23] or women [24]. However, none of these studies specifically evaluated the risk of CKD according to different time windows of exposure, although they evaluated previous exposure to NSAIDs.

Moreover, large observational cohort studies, such as the Nurses’ Health Study [24] and the study on healthy male physicians [40] used questionnaires to determine exposure to NSAIDs and it is possible that recall bias affected the study results. However, in the elderly population, high cumulative NSAID exposure was associated with an increased risk of rapid CKD progression [25].

In our study, the risk of developing CKD increased by almost 70% for current users of oxicams. This increase in risk was more striking for meloxicam and piroxicam. This may be due long half-life of oxicams, for piroxicam (50 hours) and meloxicam (20 hours) in particular. The prolonged half-life results in the maintenance of relatively stable plasma concentrations throughout the day on once daily doses and to significant accumulation upon multiple dosing, as reported in the Summary of Product Characteristics (SPC) of the drugs. Both of the oxicams act by reducing prostaglandins synthesis through COX inhibition.

The long-term use of NSAIDs, such as piroxicam, has been known to result in renal injury, including renal papillary necrosis [42]. Interestingly, the highest risk of CKD was observed for piroxicam and ketorolac which are known to have also the highest risk of other serious NSAID-related side effects such as upper gastrointestinal risk [43, 44]. Renal toxicity may be a risk particularly for patients in whom renal prostaglandins play a major role in maintaining renal perfusion. In this case, the decrease in prostaglandin formation brought NSAIDs may reduce renal blood flow and result in renal decompensation [42].

In line with these considerations, we found the highest risk of CKD in long-term users of meloxicam and piroxicam even though this analysis was limited by the low number of exposed patients. It should be noted that CKD is not currently reported among the possible side effects of piroxicam or meloxicam in the SPC of the drugs. In particular, the risk of CKD increased by 28% and by 19% for each additional cumulative month of therapy with meloxicam or piroxicam within one year prior to the ID, despite the lack of statistical significance.

Our results suggest a 2.5-fold increase of CKD risk also with the short-term use of ketorolac, implying that this high-potency NSAID could facilitate progression of subclinical CKD leading to clinically manifested CKD. While many NSAIDs are indicated for chronic conditions such as osteoarthritis or rheumatoid arthritis, ketorolac is indicated only for short-term use (i.e., maximum of 5 days as oral therapy or two days as intramuscular therapy) for the treatment of moderate-severe acute pain, including post-surgical pain [45, 46]. In the past, ketorolac was also used in the long-term management of chronic cancer pain and pain of other etiologies (e.g. osteoarthritis). Over the years, there has been an increasing awareness of the risk of adverse effects with ketorolac, especially gastrointestinal bleeding or ulceration. The duration of ketorolac use was therefore restricted to just a few days of therapy [47].

According to the drug SPC, ketorolac is contraindicated in moderate to severe renal impairment (serum creatinin >442 μmol/l) and in patients at risk of renal failure due to volume depletion or dehydration. As it is a potent prostaglandin inhibitor, ketorolac should be used cautiously in patients with a history of renal disease or otherwise impaired renal function. Ketorolac-related renal toxicity has been reported in conditions associated with hypovolemia and/or renal blood flow where renal prostaglandins have an important role in maintaining kidney perfusion. In these conditions, the use of ketorolac can lead to a decrease in renal prostaglandins, increasing the risk of precipitating renal failure [48, 49].

The association between exposure to piroxicam, meloxicam or ketorolac and adjusted risk of CKD was not modified by the presence of hypertension or diabetes mellitus.

### Limitations

This study has several strengths and limitations. The methodology applied in the present study relied on a healthcare database and therefore has the associated advantages. For example, recall and selection bias are not a concern since data is recorded at the point of routine care and all GPs contribute data.

However, since the diagnoses were made by GPs rather than by nephrologists, it is possible that some CKD diagnoses may not be accurate, notwithstanding our search for specific CKD codes. For this reason outcome misclassification cannot be completely ruled out. To prevent misclassification of AKI as CKD during the case ascertainment, CKD codes were only considered to imply CKD if they were registered more than once.

It was possible to identify the precise date of CKD onset because we considered the first registration of a CKD-related healthcare service to act as a proxy for the former. However, we considered at least three months and even longer time windows to take into account the possible lag time between clinical manifestation of impaired renal function and CKD registration. It was not possible to identify the stage of CKD for the majority of CKD patients as the laboratory data necessary to stage the disease is not available from Arianna and registered CKD codes rarely refer to staged CKD.

However, CKD stage-specific codes were reported for 339 (17.0%) patients with CKD and a sensitivity analysis restricted to these patients confirmed main results. Since outpatient prescription data was used, it cannot be definitively said that the prescriptions were ultimately filled and the medication taken. However, since prescription NSAIDs are mainly used as anti-inflammatory drugs in chronic diseases such as arthritis, it is unlikely that they were not taken.

The prescription of over-the-counter (OTC) NSAIDs cannot be traced using this database, as such drugs are not reimbursed by the Italian National Health Service. This may give rise to a potential under-estimation of NSAID exposure concerning NSAIDs with OTC status in Italy (e.g. ibuprofen, acetylsalicylic acid, ketoprofen lysine salt, and others). However, NSAIDs used as OTC drugs contain much lower dosage of active ingredient as compared to prescribed NSAIDs and the dose-effect response, which may play a role in NSAID-associated CKD occurrence, is therefore likely to be much less significant than for prescription NSAIDs.

## Conclusions

While the acute kidney-related effects associated with the use of NSAIDs have been extensively investigated, the chronic kidney-related effects of NSAIDs are not. However, the findings of this study show a differential risk of chronic renal damage across individual NSAIDs. Long-term exposure to NSAIDs with the longest half-life, such as oxicams, is associated with an increased risk of CKD. Likewise, short-term use of ketorolac is also associated with an increased risk of CKD, probably acting as a trigger of renal function deterioration in patients with subclinical CKD.

## Supporting Information

S1 Table Proportion of cases and controls using none, one or more than one NSAID over the different time windows. ID = index date; NSAID = non-steroidal anti-inflammatory drug.
(DOCX)
