# Supplementary material for: Association of Individual Non-Steroidal Anti-Inflammatory Drugs and Chronic Kidney Disease: A Population-Based Case Control Study
Source: PLoS One. 2015 Apr 16;10(4):e0122899. doi: 10.1371/journal.pone.0122899 (PMC4399982; doi:10.1371/journal.pone.0122899)
Supplement: S1 Table — ID = index date; NSAID = non-steroidal anti-inflammatory drug. (DOCX) [file pone.0122899.s001.docx]

|  | **Cases (N= 1,989)** | | | **Controls (N= 7,906)** | | |
| --- | --- | --- | --- | --- | --- | --- |
| **Time windows (prior to ID)** | **One NSAID use** | **More than one NSAID use** | **Non use** | **One NSAID use** | **More than one NSAID use** | **Non use** |
|  | **N (%)** | **N (%)** | **N (%)** | **N (%)** | **N (%)** | **N (%)** |
| 90 days | 397 (20.0) | 102 (5.1) | 1,490 (74.9) | 1,367 (17.3) | 322 (4.1) | 6,217 (78.6) |
| 180 days | 479 (24.1) | 220 (11.0) | 1,290 (64.9) | 1,820 (23.0) | 694 (8.8) | 5,392 (68.2) |
| 365 days | 540 (27.1) | 401 (20.2) | 1,048 (52.7) | 2,139 (27.1) | 1,370 (17.3) | 4,397 (55.6) |

**S1 Table.** Proportion of cases and controls using none, one or more than one NSAID over the different time windows.
